# CD40 expression in the hippocampus and its role in blood-brain barrier permeability, neutrophil infiltration and oxidative stress: implication for brain damage associated with sepsis in rats

**DOI:** 10.1186/cc12976

**Published:** 2013-11-05

**Authors:** Monique Michels, Lucinéia G Danielski, Andriele A da Silva Vieira, Luiz Carlos Vieira, Drielly Florentino, André Laurenano, Francielle Mina, Francieli Vuolo, Dhébora M Dall'Igna, Letícia Galant, João Quevedo, Felipe Dal-Pizzol, Fabrícia Petronilho

**Affiliations:** 1 Laboratório de Fisiopatologia Clínica e Experimental, UNISUL, Tubarão, SC, Brazil; 2 Laboratório de Neurociências, UNESC, Criciúma, SC, Brazil; 3 Laboratório de Fisiopatologia Experimental, UNESC, Criciúma, SC, Brazil

## Background

Sepsis is a clinical condition resulting from the excessive inflammatory response of the host against an infectious agent and is associated with high morbidity and mortality in patients in ICUs. In sepsis the brain can be targeted, associated with mental damage and decline, impaired attention, disorientation, delirium and coma. It has been seen that the permeability of the blood-brain barrier (BBB) is associated with septic encephalopathy, allowing cell infiltration and increased oxidative stress. Accordingly, such events can be potentiated through the involvement of molecules that when activated perpetuate the inflammatory response and the breaking of the BBB, and it is possible to postulate that the CD40 molecule may be involved by being under increased expression in microglia in inflammatory events occurring systemically. The aim of this study therefore is to evaluate the role of CD40 in the breakdown of the BBB, cell infiltration and oxidative damage in the brain of rats with sepsis.

## Materials and methods

Male Wistar rats were subjected to cecal ligation and puncture (CLP) to induce sepsis. The animals (*n *= 10) were divided into sham, CLP, CLP + 1 ng, CLP + 10 ng and CLP + 100 ng anti-CD40 antibody administered intracerebroventricularly. The rats were killed at 24 hours for assessment of oxidative damage in lipids (TBARS), damage to proteins by protein carbonylation, nitrite/nitrate concentration (NO), myeloperoxidase (MPO) and breakdown of the BBB. The other group was subjected to CLP and after 24 hours they were killed and the hippocampus removed to analyse expression of CD40 and CD40L by western blotting. Data were evaluated by ANOVA and *post-hoc *Tukey test with significance *P *< 0.05.

## Results

Our results show that in the most effective dose of 100 ng/kg anti-CD40 showed a decrease in the breakdown of the BBB, MPO, nitrite/nitrate concentration and TBARS. A dose of 1 ng/kg was effective only in the reduction of nitrite/nitrate concentration and 10 ng/kg was not effective in TBARS and carbonyl. Western blotting analysis showed increased expression of CD40 and CD40L in CLP animals when compared with sham.

## Conclusions

Modulation of the levels of CD40 may represent a potential therapeutic target in sepsis.

